# Field output correction factors and perturbation factor analysis for novel SunSILICON and SunSILICON P silicon diode detectors

**DOI:** 10.1002/acm2.70632

**Published:** 2026-05-31

**Authors:** Andreas A. Schönfeld, Mohamad Alissa, David Towle, Andy Murray, Ann‐Britt Schönfeld, Teresa J. Anders, Erik Alquist, Olivier Evrard, Chuck Rosenwald, Jeff Hildreth, Veronika Flatten, Gerhard Wessing, Charbel Habib, Damian Czarnecki

**Affiliations:** ^1^ Sun Nuclear Corp. Melbourne Florida USA; ^2^ CDT‐West—Centrum für Diagnostik und Therapie Cologne Germany; ^3^ Ärztepartnerschaft Radiologie Vechta Vechta Germany; ^4^ Universität Bremen Bremen Germany; ^5^ Mirion Technologies Olen Belgium; ^6^ MyMichigan Health Midland Michigan USA; ^7^ Institut für Medizinische Physik und Strahlenschutz, Technische Hochschule Mittelhessen Gießen Germany

**Keywords:** dosimetry, field output correction factors, field output factors, silicon detectors, small photon field dosimetry

## Abstract

**Background:**

Field output factor measurements of clinical linear accelerators require detector‐specific corrections, due to the introduction of fluence perturbation effects by non‐water‐equivalent components of applied detectors.

**Purpose:**

This study aims to determine field output correction factors for novel shielded and unshielded silicon diode detectors in high energy photon fields. Special emphasis is placed on understanding the influence of perturbation factors and evaluating the suitability of these detectors across a wide range of field sizes.

**Methods:**

Field output correction factors for the silicon diodes SunSILICON and SunSILICON P were determined through experimental measurements conducted at four distinct sites. Monte Carlo‐based models of the diode detectors were developed to calculate field output correction factors using the EGSnrc code system. In addition, these silicon diode models enabled a detailed analysis of perturbation factors for field sizes from 0.6 cm up to 40 cm.

**Results:**

Experimentally determined and Monte Carlo calculated field output correction factors are in good agreement for the detectors investigated. The perturbation factor analysis demonstrated a strong field size dependence of the fluence perturbation factor for silicon.

**Conclusion:**

This is the first study to systematically characterize the SunSILICON diode detector family using both simulation and measurement. The findings confirm that both shielded and unshielded designs are suitable for clinical dosimetry in photon beams, requiring only minor corrections. The shielding of the SunSILICON P diode enables accurate field output factor measurements across a broad range of field sizes, establishing its utility in modern radiotherapy applications.

## 1 INTRODUCTION

In modern external photon‐beam radiation therapy, high‐dose conformity can be achieved through the superposition of small, dynamically modulated beamlets, typically delivered from one or multiple rotational planes. Stereotactic radiotherapy techniques for targeting sub‐centimeter lesions have also become increasingly common.

The commissioning process and quality assurance of such beamlets are challenging, since small field photon dosimetry is characterized by secondary electron disequilibrium and partial source occlusion.^1^, ^2^ These small field conditions amplify the impact of radiation fluence perturbations, which are introduced by any detector components that are not water equivalent.^3^ While suitable detectors need to be sufficiently small,^1^ their dosimetric behavior with respect to the radiation field size is governed not only by the physical dimensions of the sensitive volume,^4^, ^5^, ^6^ but also by the choice of the detection material^6^, ^7^, ^8^, ^9^, ^10^, ^11^, ^12^, ^13^, ^14^, ^15^, ^16^ and the construction of the detector housing.^6^, ^17^, ^18^, ^19^ Since the measurement of the linear accelerator (linac) output with respect to field size is a key task in the linac's commissioning process and quality assurance,^1^, ^20^ the characteristic dosimetric behavior of a specific detector needs to be well‐determined so that it can be corrected.^1^, ^4^, ^5^, ^13^, ^21^, ^22^, ^23^, ^24^, ^25^, ^26^, ^27^, ^28^, ^29^, ^30^ Dedicated small field dosimetry protocols provide detector‐type specific corrections for commonly used detectors, such as the field output correction factor $k_{Q_{\mathrm{clin}},Q_{\mathrm{ref}}}^{f_{\mathrm{clin}},f_{\mathrm{ref}}}$.^1^, ^31^, ^32^, ^33^, ^34^

Silicon diode detectors are commonly used for small field photon dosimetry measurements, due to their compact size and high signal strength compared to air‐filled ionization chambers. While the energy required to produce an ion pair in air is 33.97 eV,^35^ only about 3.62–3.86 eV are necessary to produce an electron‐hole pair in silicon.^36^ In combination with the enhanced density of silicon, a suitable detector sensitivity can be achieved with very small sensitive volumes.^37^

However, a thorough analysis of silicon as a detector material reveals that the deviating mean excitation energy (*I*‐value; $I_{w}=78$ eV vs. $I_{Si}=173$ eV^38^) and electron density produce a complex, characteristic detector behavior common to all silicon‐based detectors, which has been thoroughly discussed in literature.^7^, ^8^, ^9^, ^10^, ^11^, ^12^, ^14^ Consequently, field output correction factor differences among silicon‐based detectors are primarily caused by fluence perturbations introduced by non‐water‐equivalent components of the detector housings.^6^, ^8^, ^13^, ^22^, ^39^ Silicon‐based detectors with near‐water‐equivalent housings are often referred to as “unshielded” detectors.^40^, ^41^, ^42^, ^43^, ^44^, ^45^, ^46^

Due to silicon's enhanced cross‐section for low‐energy photons, the varying occurrence of energy‐degraded Compton scatter with respect to field size and depth in water^44^, ^47^, ^48^ can affect the accuracy of relative dosimetry measurements.^25^, ^41^, ^44^, ^48^, ^49^ Therefore, the use of unshielded silicon diodes is typically recommended in field sizes of 10 cm or less. The new 1048 SunSILICON detector (Sun Nuclear Corp, Melbourne, USA) investigated in this study follows an unshielded detector design^42^ and is dedicated for dosimetry in the smallest photon fields with a specified field size range from 0.4 cm up to 10 cm. Field sizes are expressed as the width of the square field, since only square fields were used in this study.

The over‐response to low‐energy Compton scatter in relative dosimetry measurements can be mitigated by adding a shield to the diode design, which is typically composed of high‐Z materials to maximize the photoelectric effect cross section.^8^ This can broaden the applicable range of field sizes to large photon fields, the maximum field size of which is determined by the quality of the shield design.^41^, ^49^ The addition of high‐Z materials, however, also enhances perturbation effects in small field sizes.^8^ Since the suitability of a detector for field output factor measurements is determined by a correction threshold of 5%, the addition of a shield can lead to the specification of a minimum field size.^1^ The new 1049 SunSILICON P detector (Sun Nuclear Corp.) studied here follows a shielded detector design^42^ and is dedicated for small to large photon field dosimetry with a specified field size range of 2–40 cm.

The goal of this study is to determine the field output correction factors for SunSILICON and SunSILICON P and to characterize the underlying perturbation effects, both by means of Monte Carlo simulations and measurement.

## 2 MATERIAL AND METHODS

### 2.1 Monte Carlo simulation settings

Monte Carlo simulations were performed using the EGSnrc code system^50^ to calculate the absorbed dose to water in a water phantom, as well as the dose deposition within the sensitive volume of two different silicon detectors. The detector simulation was defined in such a way that it mirrors the experimental measurement conditions.

#### 2.1.1 Radiation source

The particle transport within an Elekta Synergy (Elekta AB, Stockholm, Sweden) 6 MV and a Varian Clinac (Varian Medical Systems, Inc., Palo Alto, USA) 10 MV linear accelerator (linac) head model was simulated using BEAMnrc from the EGSnrc code system to compute accurate clinical radiation fields. The linac head models had been validated and utilized in previously published studies.^51^, ^52^ In this work, the radiation field size of the linac model was varied by adjusting the multi‐leaf collimator and jaws according to the corresponding linac settings in the experimental study. The resulting dosimetric field size was determined by calculating the full width at half maximum (FWHM) in lateral beam profile simulations at 10 cm depth and a source‐to‐surface distance of 90 cm. To calculate bremsstrahlung production more efficiently, the variance reduction technique directional bremsstrahlung splitting (DBS) with a splitting factor of 1500 was used.^53^ The splitting radius of the DBS technique was adjusted to be equal to the longest side of the radiation field at the isocenter. For instance, a field size of 10 cm corresponded to a splitting radius of 10 cm. Additionally, to save computing time, the transport and particle production threshold energies for electrons and photons in the linac head geometry were set to ECUT = AE = 521 keV and PCUT = AP = 10 keV, respectively—values commonly used in EGSnrc. Using BEAMnrc, phase‐space files were generated to serve as detailed, physics‐based source descriptions for subsequent dose calculations within the water phantom.

#### 2.1.2 Dose calculation

**FIGURE 1:**
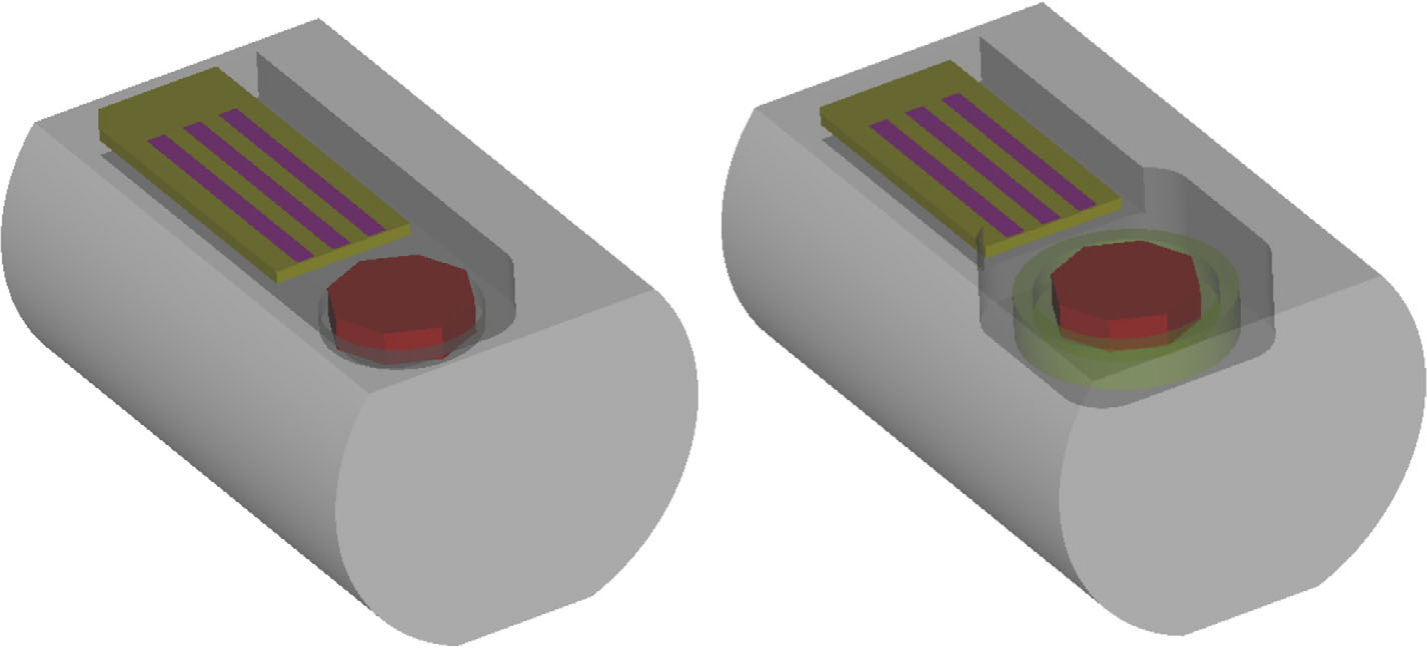
Monte Carlo models of the silicon diode detectors SunSILICON (left) and SunSILICON P (right). Colors are used to depict different parts of the diode detectors. For better clarity, some elements are shown as semi‐transparent. Image not to scale. Not all details are shown.

Detailed models of the two silicon diode detectors 1048 SunSILICON and 1049 SunSILICON P were created using the egs++ class library (Figure 1). The Monte Carlo code egs_chamber was used to calculate the particle transport within the water phantom and detectors.^54^ Phase‐space files generated in BEAMnrc were used as a radiation source.

The total number of particle histories were set to achieve statistical uncertainty below 0.15% in regions of interest. To reduce computation time, the variance reduction techniques Russian roulette, photon cross section enhancement (XCSE), and intermediate phase space scoring were applied.

The transport and particle production threshold energies for electrons and photons in the water phantom and detector models were set to ECUT = AE = 512 keV and PCUT = AP = 1 keV, respectively, to achieve greater accuracy.

#### 2.1.3 Monte Carlo calculations of field output correction factors

The field output factor $\Omega_{Q_{\mathrm{clin}},Q_{\mathrm{ref}}}^{f_{\mathrm{clin}},f_{\mathrm{ref}}}$ for the clinical field $f_{\mathrm{clin}}$ and beam quality $Q_{\mathrm{clin}}$ was calculated using Equation 1, using the formalism introduced by Alfonso et al.^55^

(1) $$\Omega_{Q_{\mathrm{clin}},Q_{\mathrm{ref}}}^{f_{\mathrm{clin}},f_{\mathrm{ref}}}=\frac{D_{w,Q_{\mathrm{clin}}}^{f_{\mathrm{clin}}}}{D_{w,Q_{\mathrm{ref}}}^{f_{\mathrm{ref}}}},$$

$D_{w,Q_{\mathrm{clin}}}^{f_{\mathrm{clin}}}$ and $D_{w,Q_{\mathrm{ref}}}^{f_{\mathrm{ref}}}$ correspond to the absorbed dose to water in the clinical field $f_{\mathrm{clin}}$ of beam quality $Q_{\mathrm{clin}}$ and in the reference field $f_{\mathrm{ref}}$ of quality $Q_{\mathrm{ref}}$, respectively.

To calculate the absorbed dose to water using Monte Carlo simulations, the dose ${\bar{D}}_{w,Q_{\mathrm{clin}}}^{f_{\mathrm{clin}}}$ is typically calculated within a small scoring volume, over which the energy deposition is averaged. This approach serves as an approximation of the point dose $D_{w,Q_{\mathrm{clin}}}^{f_{\mathrm{clin}}}∼\;{\bar{D}}_{w,Q_{\mathrm{clin}}}^{f_{\mathrm{clin}}}$. In this work, a cylindrical volume was chosen to approximate a point dose (see Appendix). The cylinder was placed symmetrically along the beam axis at a water depth of 10 cm to calculate the absorbed dose to water. The height h of the cylinder was 0.2 cm, and its radius r was fixed at 0.2 cm for field sizes greater than 2 cm.

However, in smaller radiation fields, even very small scoring volumes can introduce a volume‐averaging effect, potentially leading to a systematic bias in the estimated dose and field output factor calculations. Further reduction of the cylindrical scoring volume is limited by the calculation time necessary to reach the desired statistical uncertainty. Nonetheless, it can be assumed that as the radius r of the cylindrical scoring volume decreases, the mean absorbed dose ${\bar{D}}_{w,Q_{\mathrm{clin}}}^{f_{\mathrm{clin}}}\left(r\right)$ approaches the point dose $D_{w,Q_{\mathrm{clin}}}^{f_{\mathrm{clin}}}$. For a relatively small range of cylinder radii, it may be assumed that the dose ${\bar{D}}_{w,Q_{\mathrm{clin}}}^{f_{\mathrm{clin}}}\left(r\right)$ decreases with increasing radius r according to the following equation:

(2) $${\bar{D}}_{w,Q_{\mathrm{clin}}}^{f_{\mathrm{clin}}}\;\left(r\right)=a\;r^{2}+D_{w,Q_{\mathrm{clin}}}^{f_{\mathrm{clin}}},$$

The absorbed dose to water $D_{w,Q_{\mathrm{clin}}}^{f_{\mathrm{clin}}}$ as well as the parameter a from 2 were determined by fitting to the calculated ${\bar{D}}_{w,Q_{\mathrm{clin}}}^{f_{\mathrm{clin}}}\left(r\right)$ for varying radii r. This extrapolation method allows for the estimation of the point dose $D_{w,Q_{\mathrm{clin}}}^{f_{\mathrm{clin}}}$ by analyzing how the average dose changes with scoring volume size. See Appendix for details.

The field output correction factor $k_{Q_{\mathrm{clin}},Q_{\mathrm{ref}}}^{f_{\mathrm{clin}},f_{\mathrm{ref}}}$, as defined in the AAPM TG 155^31^ and IAEA TRS 483 dosimetry protocol using 10 cm as reference field size,^1^ was calculated with Monte Carlo simulations under the assumption that the detector signal $M_{Q_{\mathrm{clin}}}^{f_{\mathrm{clin}}}$ is directly proportional to the dose deposited within the sensitive volume of the detector ${\bar{D}}_{\det,Q_{\mathrm{clin}}}^{f_{\mathrm{clin}}}$:

(3) $$\Omega_{Q_{\mathrm{clin}},Q_{\mathrm{ref}}}^{f_{\mathrm{clin}},f_{\mathrm{ref}}}=k_{Q_{\mathrm{clin}},Q_{\mathrm{ref}}}^{f_{\mathrm{clin}},f_{\mathrm{ref}}}\;\frac{{\bar{D}}_{\det,Q_{\mathrm{clin}}}^{f_{\mathrm{clin}}}}{{\bar{D}}_{\det,Q_{\mathrm{ref}}}^{f_{\mathrm{ref}}}}=k_{Q_{\mathrm{clin}},Q_{\mathrm{ref}}}^{f_{\mathrm{clin}},f_{\mathrm{ref}}}\;\frac{M_{Q_{\mathrm{clin}}}^{f_{\mathrm{clin}}}}{M_{Q_{\mathrm{ref}}}^{f_{\mathrm{ref}}}},$$

Then, the following equation can be derived to determine the field output factor $k_{Q_{\mathrm{clin}},Q_{\mathrm{ref}}}^{f_{\mathrm{clin}},f_{\mathrm{ref}}}$ using Monte Carlo simulations:

(4) $$k_{Q_{\mathrm{clin}},Q_{\mathrm{ref}}}^{f_{\mathrm{clin}},f_{\mathrm{ref}}}=\frac{{\bar{D}}_{w,Q_{\mathrm{clin}}}^{f_{\mathrm{clin}}}/{\bar{D}}_{\det,Q_{\mathrm{clin}}}^{f_{\mathrm{clin}}}}{{\bar{D}}_{w,Q_{\mathrm{ref}}}^{f_{\mathrm{ref}}}/{\bar{D}}_{\det,Q_{\mathrm{ref}}}^{f_{\mathrm{ref}}}},$$

#### 2.1.4 Perturbation factor

Using the formalism described by Bouchard et al.,^6^, ^56^ the field output correction factor $k_{Q_{\mathrm{clin}},Q_{\mathrm{ref}}}^{f_{\mathrm{clin}},f_{\mathrm{ref}}}$ can be expressed in terms of the mean restricted stopping power ratio of water and silicon $s_{w,det}$, and the product of several multiplicative perturbation factors $p_{i}$.

(5) $$k_{Q_{\mathrm{clin}},Q_{\mathrm{ref}}}^{f_{\mathrm{clin}},f_{\mathrm{ref}}}=\frac{{\left[s_{w,\mathrm{Si}}\prod_{i}p_{i}\right]}_{Q_{\mathrm{clin}}}^{f_{\mathrm{clin}}}}{{\left[s_{w,\mathrm{Si}}\prod_{i}p_{i}\right]}_{Q_{\mathrm{ref}}}^{f_{\mathrm{ref}}}},$$

While this formalism has been applied to solid state detectors,^13^, ^22^, ^39^ the factorization of individual perturbation components is not always consistent across different studies.^7^, ^8^, ^9^, ^10^, ^11^, ^13^, ^22^ Following the concept of the perturbation analysis for ionization chambers, a series of diode detector models was created in which the detector components were gradually removed. This resulted in a series of scoring volumes in which the dose was calculated, where the listed scoring volumes are surrounded by water:

${\bar{D}}_{\det}$
: Average dose absorbed in the sensitive volume of the silicon chip in the detector housing, see equation 3.
${\bar{D}}_{\mathrm{chip}}$
: Average dose absorbed in the sensitive volume of the silicon chip in absence of the detector housing.
${\bar{D}}_{\mathrm{sens},\mathrm{Si}}$
: Average dose absorbed in a silicon disc matching the dimensions of the silicon detector's sensitive volume.
${\bar{D}}_{\mathrm{sens},w*}$
: Average dose absorbed in a disc matching the dimensions of the silicon detector's sensitive volume and consisting of modified water (w*) with the density of silicon (2.33 g/cm^3^).
${\bar{D}}_{\mathrm{sens},w}$
: Average dose absorbed in a water cavity matching the dimensions of the silicon detector's sensitive volume.
$D_{w}$
: Absorbed dose in an infinitesimally small volume of water, see equation 2.

Using these dose values, the following perturbation factors can be calculated to quantify the perturbation caused by different detector components:

(6) $$p_{h}=\frac{{\bar{D}}_{\mathrm{chip}}}{{\bar{D}}_{\det}},$$

(7) $$p_{\mathrm{chip}}=\frac{{\bar{D}}_{\mathrm{sens},\mathrm{Si}}}{{\bar{D}}_{\mathrm{chip}}},$$

(8) $$s_{w,\mathrm{Si}}\;p_{\mathrm{fl}}=\frac{{\bar{D}}_{\mathrm{sens},w*}}{{\bar{D}}_{\mathrm{sens},\mathrm{Si}}},$$

(9) $$p_{\rho }=\frac{{\bar{D}}_{\mathrm{sens},w}}{{\bar{D}}_{\mathrm{sens},w*}},$$

(10) $$p_{\mathrm{vol}}=\frac{D_{w}}{{\bar{D}}_{\mathrm{sens},w}},$$

**FIGURE 2:**
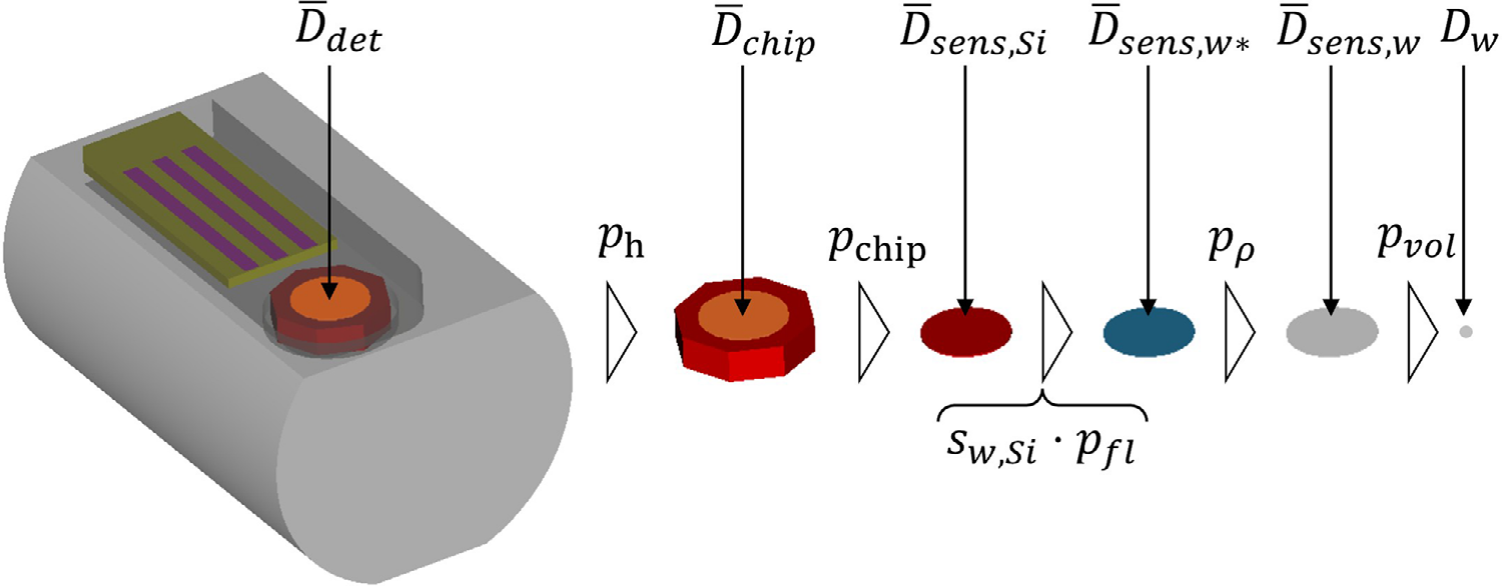
Schematic representation of a sequence of perturbation factors for a diode detector. The average dose in each cavity from which an arrow originates can be corrected by the perturbation factor next to the arrow to the average dose in the cavity to which the arrow points. While only the unshielded housing variant is shown on the left, an analogue decomposition was done for the shielded variant shown in the right panel of Figure 1. $p_{h}$ is then expressed as $p_{h,\mathrm{unsh}.}$ and $p_{h,\mathrm{sh}.}$ for the unshielded and shielded variant, respectively.

Using Equations (6), (7), (8), (9), (10), the perturbation of each component of the silicon diode detector can be quantified separately. Figure 2 schematically illustrates the procedure used to determine the perturbation factors. The change of the electron fluence caused by the silicon diode housing is considered by the perturbation factor $p_{h}$, where $p_{h,unsh.}$ and $p_{h,sh.}$ correspond to the unshielded and shielded housing variants (see Figure 1). The perturbation factor $p_{chip}$ describes the change in the absorbed dose in the silicon chip caused by the non‐sensitive volume of the silicon chip. The change of the atomic composition of the sensitive volume from silicon to water has two principal effects on the absorbed dose: Firstly, the change in photon interaction cross sections between silicon and water leads to a difference in the electron fluence, especially in the low energy range (< 0.2 MeV)—described by the fluence perturbation factor $p_{\mathrm{fl}}$. Secondly, the variation in dose deposition by electrons due to the difference in mass stopping powers of silicon and water—described by the mass stopping power ratio water to silicon $s_{w,Si}$. The perturbation resulting from increased density is described by the density perturbation factor $p_{\rho }$, corresponding to $p_{\rho -}$ discussed in Fenwick et al. 2013.^11^ The perturbation factor $p_{vol}$ quantifies the deviation introduced when the absorbed dose is measured over the finite sensitive volume of the detector, rather than at a point in the water phantom.

In relative dosimetry, such as the determination of field output factors $\Omega_{Q_{\mathrm{clin}},Q_{\mathrm{ref}}}^{f_{\mathrm{clin}},f_{\mathrm{ref}}}$, the relative variation in perturbation factors between the reference and clinical radiation beam is relevant, due to the significant alteration of the fluence spectrum the detector is exposed to.^48^ For this reason, all perturbation factors in this study were examined relative to those under reference conditions.

### 2.2 Experimental setup

The experimental determination of field output correction factors $k_{Q_{\mathrm{clin}},Q_{\mathrm{ref}}}^{f_{\mathrm{clin}},f_{\mathrm{ref}}}$ for SunSILICON and SunSILICON P follows Equation 3, where

(11) $$k_{Q_{\mathrm{clin}},Q_{\mathrm{ref}}}^{f_{\mathrm{clin}},f_{\mathrm{ref}}}=\Omega_{Q_{\mathrm{clin}},Q_{\mathrm{ref}}}^{f_{\mathrm{clin}},f_{\mathrm{ref}}}\;\cdot \frac{M_{Q_{\mathrm{ref}}}^{f_{\mathrm{ref}}}}{M_{Q_{\mathrm{clin}}}^{f_{\mathrm{clin}}}},$$

In Equation 11, $M_{Q_{\mathrm{ref}}}^{f_{\mathrm{ref}}}/M_{Q_{\mathrm{clin}}}^{f_{\mathrm{clin}}}$ was measured with SunSILICON and SunSILICON P, respectively. $\Omega_{Q_{\mathrm{clin}},Q_{\mathrm{ref}}}^{f_{\mathrm{clin}},f_{\mathrm{ref}}}$ was measured with well‐characterized reference detectors for which consensus field output correction factors $k_{Q_{\mathrm{clin}},Q_{\mathrm{ref}}}^{f_{\mathrm{clin}},f_{\mathrm{ref}}}$ are available and those corrections are small.

The reference detectors chosen for field sizes of 10 cm and less were Standard Imaging's Exradin W1 scintillation detector and a PTW 60019 microDiamond detector. Corresponding $k_{Q_{\mathrm{clin}},Q_{\mathrm{ref}}}^{f_{\mathrm{clin}},f_{\mathrm{ref}}}$ values were taken from TRS 483^1^ and applied according to Equation 3.

Two different detector types were chosen to cross‐validate $\Omega_{Q_{\mathrm{clin}},Q_{\mathrm{ref}}}^{f_{\mathrm{clin}},f_{\mathrm{ref}}}$, due to uncertainty contributions associated with the respective experimental setup as well as the detector‐specific $k_{Q_{\mathrm{clin}},Q_{\mathrm{ref}}}^{f_{\mathrm{clin}},f_{\mathrm{ref}}}$ values^1^ in small field sizes. To further enhance confidence in measured $\Omega_{Q_{\mathrm{clin}},Q_{\mathrm{ref}}}^{f_{\mathrm{clin}},f_{\mathrm{ref}}}$ data, additional validation measurements were taken with a larger set of detectors. Detectors selected for validation measurements only differ by having larger correction values $k_{Q_{\mathrm{clin}},Q_{\mathrm{ref}}}^{f_{\mathrm{clin}},f_{\mathrm{ref}}}$.

Compact‐type ionization chambers or Farmer‐type ionization chambers were used as reference for field sizes of 10 cm and above to minimize uncertainty contributions from cable and detector stem effects.

Measurements were performed using various linear accelerators with and without flattening filter, with nominal energies ranging from 4 MV to 25 MV. 4 MV and 25 MV measurements were conducted at the German national metrology institute PTB Braunschweig (Elekta Versa HD). Other measurement sites included Sun Nuclear's headquarter in Melbourne, FL, (Varian TrueBeam), MyMichigan Health, Saginaw, MI, (Varian TrueBeam) and Radiologie Vechta, Vechta, Germany (Elekta Versa HD).

An overview of the equipment used for experimental determination of field output correction factors $k_{Q_{\mathrm{clin}},Q_{\mathrm{ref}}}^{f_{\mathrm{clin}},f_{\mathrm{ref}}}$ is provided in Table 1. Notably, the data was taken independently by the co‐authors using four different SunSILICON and SunSILICON P detectors, respectively. Water phantoms as well as reference detectors were chosen based on the availability at each respective site.

**TABLE 1:** Equipment used for experimental determination of field output correction factors $k_{Q_{\mathrm{clin}},Q_{\mathrm{ref}}}^{f_{\mathrm{clin}},f_{\mathrm{ref}}}$.

Linear accelerators	Elekta Versa HD Varian TrueBeam
Water phantoms	Sun Nuclear SunSCAN 3D PTW BeamSCAN IBA BluePhantom 2
Reference detectors (field sizes ≤ 10 cm)	PTW 60019 microDiamond Standard Imaging Exradin W1
Reference detectors (field sizes ≥ 10 cm)	Sun Nuclear 1047 SNC600c PTW 30013
Detectors under investigation	Sun Nuclear 1048 SunSILICON Sun Nuclear 1049 SunSILICON P
Validation detectors	Sun Nuclear 1118 EDGE Detector Sun Nuclear 1041 SNC125c PTW 31021 Semiflex 3D IBA CC04 IBA CC13

Detectors were set up on the central beam axis at a 10 cm depth and 90 cm source‐to‐surface distance, following the manufacturers' setup guidelines for water phantoms and detectors and the recommendations of TRS 483.^1^ Beam centering was performed for each individual small field measurement with a field size of 3 cm or less, and dosimetric field sizes at measurement depth were determined by measuring the FWHM in lateral beam scans.

### 2.3 Data fitting

Monte Carlo simulated and experimentally determined data for SunSILICON was combined and fitted using the fitting function described in TRS 483^1^:

(12) $$k_{Q_{\mathrm{clin}},Q_{\mathrm{ref}}}^{f_{\mathrm{clin}},f_{\mathrm{ref}}}\;\left(S\right)=\frac{1+e^{-\frac{10-a}{b}}}{1+e^{-\frac{S-a}{b}}}+c\cdot \left(S-10\right),$$

Where, a, b, and c are the fitting parameters and S describes the square field size in cm. The data for SunSILICON P, however, extends to a field size of 40 cm, for which the TRS 483 fit is not suitable. Thus, Monte Carlo simulated and experimentally determined data was combined and fitted using a power function:

(13) $$k_{Q_{\mathrm{clin}},Q_{\mathrm{ref}}}^{f_{\mathrm{clin}},f_{\mathrm{ref}}}\;\left(S\right)=a\cdot S^{b}+c,$$

Experimental data and Monte Carlo results were equally weighted to reduce data size bias.

## 3 RESULTS

### 3.1 Field output correction factors

**FIGURE 3:**
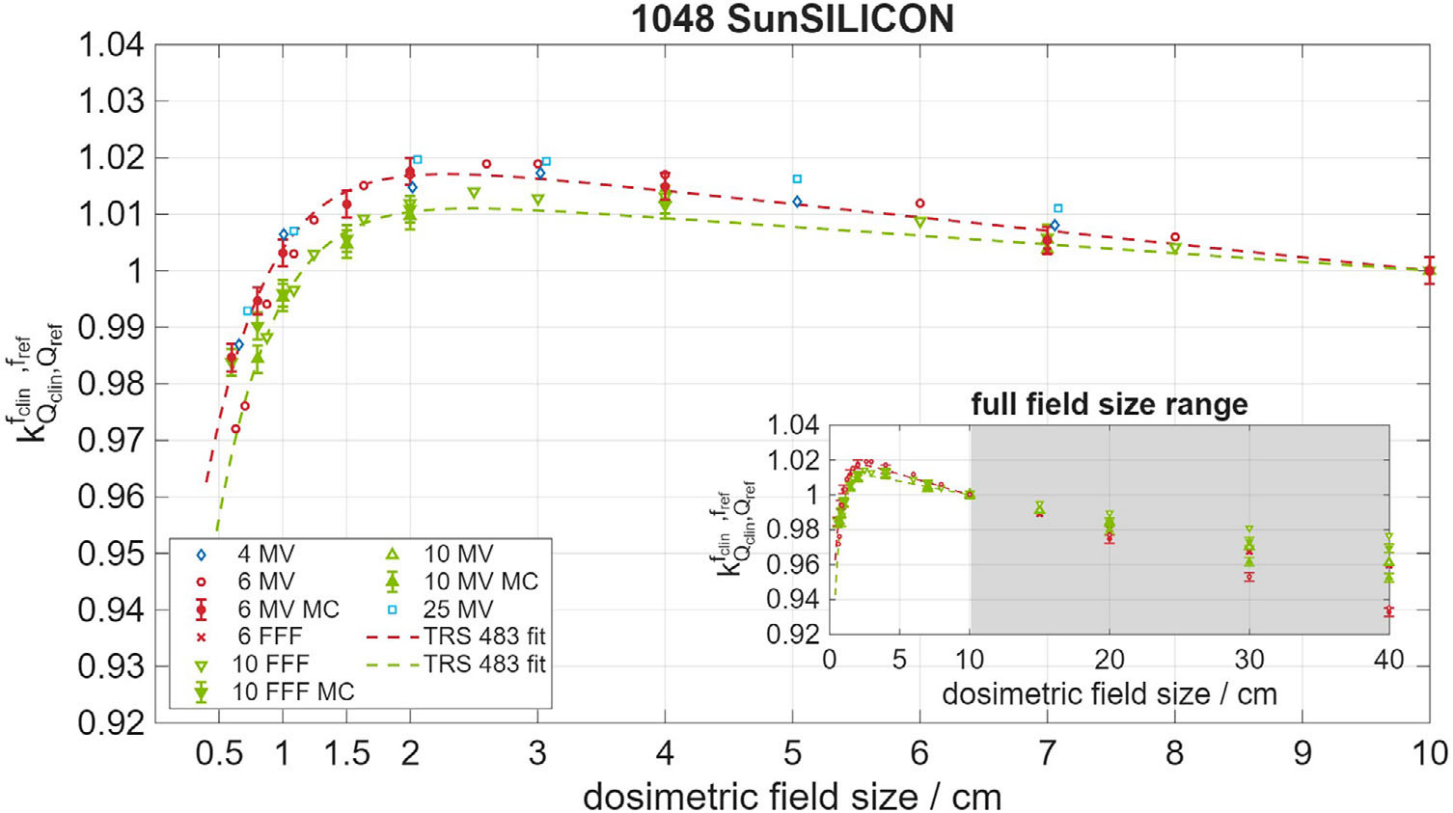
Measured and Monte Carlo simulated field output correction factors $k_{Q_{\mathrm{clin}},Q_{\mathrm{ref}}}^{f_{\mathrm{clin}},f_{\mathrm{ref}}}$ for 1048 SunSILICON for various photon beam energies. 6 MV and 10 FFF energies were measured on a Varian TrueBeam linear accelerator. 4 MV and 25 MV energies were measured on an Elekta Versa HD linear accelerator. The shaded area indicates field sizes outside of manufacturer's specifications. Equation 12 was used as fitting model.

Measured and Monte‐Carlo simulated photon field output correction factors $k_{Q_{\mathrm{clin}},Q_{\mathrm{ref}}}^{f_{\mathrm{clin}},f_{\mathrm{ref}}}$ for 1048 SunSILICON agreed with a root‐mean‐square error (RMSE) of 0.0029 (6 MV, 6 FFF) and 0.0018 (10 MV, 10 FFF). $k_{Q_{\mathrm{clin}},Q_{\mathrm{ref}}}^{f_{\mathrm{clin}},f_{\mathrm{ref}}}$ values for 1048 SunSILICON range from 0.963 at 0.4 cm field size to 1.017 at 2.5 cm field size (6 MV, 6 FFF) and from 0.943 at 0.4 cm field size to 1.011 at 2.5 cm field size (10 MV, 10 FFF), see Table 2 and Figure 3.

**TABLE 2:** Field output correction factors for 1048 SunSILICON and 1049 SunSILICON P for field sizes ranging from 0.4 to 40 cm corresponding to data fits shown in Figures 3 and 4. Entries highlighted with a * are outside of the field size ranges specified for the corresponding detector model.

	Field output correction factor			
	1048 SunSILICON		1049 SunSILICON P	
Dosimetric Field Size	6 MV / 6 FFF	10 MV / 10 FFF	6 MV / 6 FFF	10 MV / 10 FFF
0.4 cm	0.963	0.943	*0.779	*0.760
0.5 cm	0.973	0.956	*0.838	*0.818
0.6 cm	0.982	0.968	*0.875	*0.855
0.7 cm	0.989	0.977	*0.900	*0.880
0.8 cm	0.995	0.984	*0.917	*0.899
0.9 cm	1.000	0.990	*0.930	*0.913
1.0 cm	1.004	0.995	*0.940	*0.924
1.2 cm	1.009	1.001	*0.954	*0.940
1.5 cm	1.014	1.007	*0.967	*0.955
2.0 cm	1.017	1.010	0.979	0.970
2.5 cm	1.017	1.011	0.985	0.978
3.0 cm	1.016	1.011	0.989	0.983
4.0 cm	1.014	1.009	0.993	0.990
5.0 cm	1.012	1.008	0.996	0.993
6.0 cm	1.009	1.006	0.997	0.996
7.0 cm	1.007	1.005	0.998	0.997
8.0 cm	1.005	1.003	0.999	0.998
9.0 cm	1.002	1.002	1.000	0.999
10 cm	1.000	1.000	1.000	1.000
15 cm	–	–	1.001	1.002
20 cm	–	–	1.002	1.003
30 cm	–	–	1.002	1.004
40 cm	–	–	1.002	1.004

**FIGURE 4:**
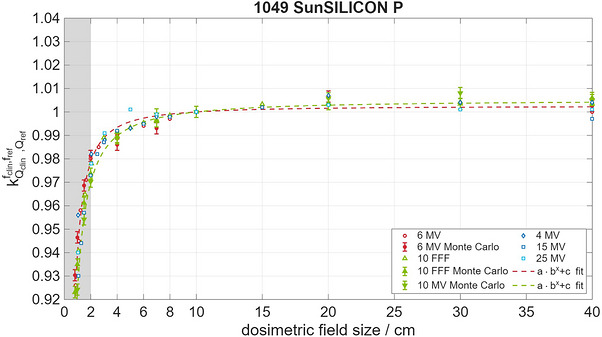
Measured and Monte Carlo simulated field output correction factors $k_{Q_{\mathrm{clin}},Q_{\mathrm{ref}}}^{f_{\mathrm{clin}},f_{\mathrm{ref}}}$ for 1049 SunSILICON P for various photon beam energies. 6 MV and 15 MV energies were measured on a Varian TrueBeam linear accelerator. 4 MV and 25 MV energies were measured on an Elekta Versa HD linear accelerator. The shaded area indicates field sizes outside of manufacturer's specifications. Equation 13 was used as fitting model.

Measured and Monte‐Carlo simulated $k_{Q_{\mathrm{clin}},Q_{\mathrm{ref}}}^{f_{\mathrm{clin}},f_{\mathrm{ref}}}$ values for 1049 SunSILICON P agreed with an RMSE of 0.0044 (6 MV, 6 FFF) and 0.0027 (10 MV, 10 FFF). $k_{Q_{\mathrm{clin}},Q_{\mathrm{ref}}}^{f_{\mathrm{clin}},f_{\mathrm{ref}}}$ values for 1049 SunSILICON P decline from 1.002 at 40 cm field size to 0.979 at 2 cm field size (6 MV, 6 FFF) and from 1.004 at 40 cm field size to 0.970 at 2 cm field size (10 MV, 10 FFF), see Table 2 and Figure 4.

Measurements obtained at 4 MV, 15 MV and 25 MV are in good agreement with the other results. Fit parameters corresponding to the applied fit models (Equations 12 and 13) using measured and Monte Carlo calculated results from the corresponding beam energies are shown in Table 3.

**TABLE 3:** Fit parameters corresponding to Table 2, Figures 3 and 4. The right‐most column shows the root‐mean‐square error (RMSE) between the fitted model and the actual data.

Detector	Energy	Function	*a*	*b*	*c*	RMSE
1048 SunSILCION	6 MV / 6 FFF	Equation 10	−8.903 ∙ 10^−1^	4.690 ∙ 10^−1^	−2.363 ∙ 10^−3^	0.0029
1048 SunSILCION	10 MV / 10 FFF	Equation 10	−7.170 ∙ 10^−1^	4.378 ∙ 10^−1^	−1.554 ∙ 10^−3^	0.0018
1049 SunSILCION P	6 MV / 6 FFF	Equation 11	−6.273 ∙ 10^−2^	−1.388	1.003	0.0044
1049 SunSILCION P	10 MV / 10 FFF	Equation 11	−8.138 ∙ 10^−2^	−1.203	1.005	0.0027

### 3.2 Perturbation factors

**FIGURE 5:**
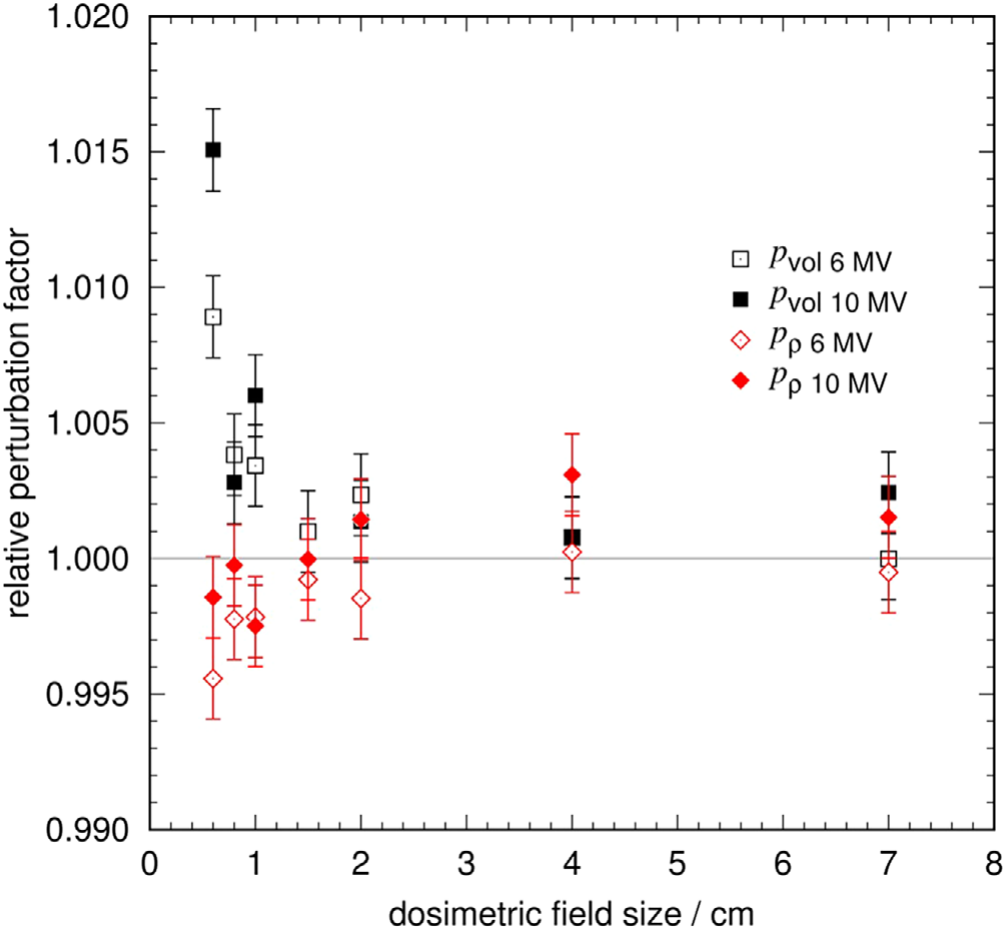
Monte Carlo calculated volume and density perturbation factors $p_{\mathrm{vol}}$ and $p_{\rho }$ normalized to the respective perturbation factors at reference field size 10 cm for the sensitive volume of the SunSILICON and SunSILICON P detector. Perturbation factors were calculated for 6 MV and 10 MV photon radiation fields, represented by open and filled symbols, respectively. The error bars represent the statistical uncertainty of the Monte Carlo calculations (1 *σ*).

**FIGURE 6:**
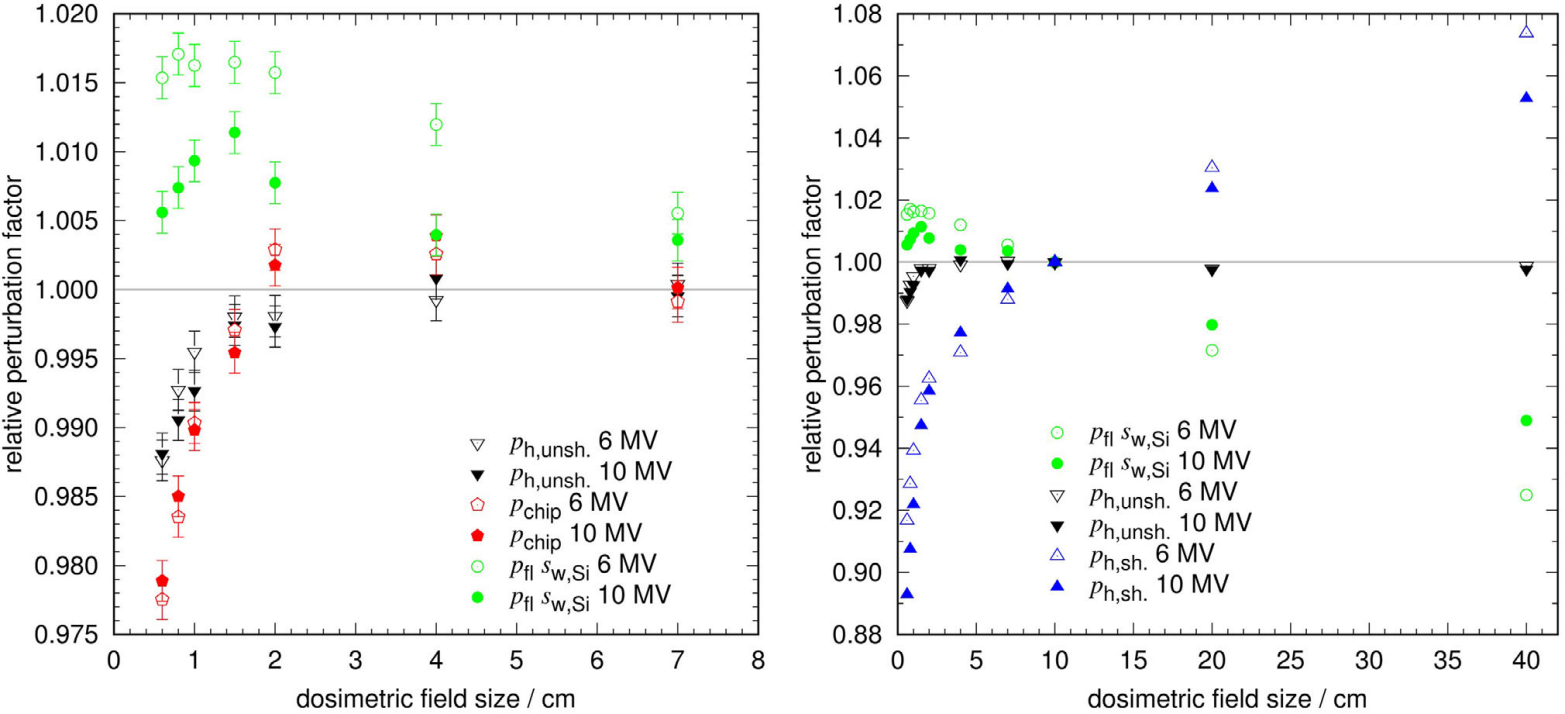
Left Panel: Monte Carlo calculated, relative perturbation factors for the detector housing of the unshielded diode SunSILICON $p_{h,\mathrm{unsh}.}$, the silicon chip $p_{\mathrm{chip}}$, and the product of the fluence perturbation $p_{\mathrm{fl}}$ and stopping power ratios water to silicon $s_{w,Si}$ as a function of field size. Right panel: Monte Carlo calculated, relative detector housing perturbation factors for the unshielded SunSILICON (downward‐pointing triangle) $p_{h,\mathrm{unsh}.}$, the shielded SunSILICON P (upward‐pointing triangle) $p_{h,\mathrm{sh}.}$ and the product of fluence perturbation and stopping power ratio water to silicon (circle) $p_{\mathrm{fl}}\;s_{w,\mathrm{Si}}$. The perturbation factors are presented for 6 MV (open symbols) and 10 MV (filled symbols) radiation fields. Refer to Figure 2 for a visual representation of the perturbation factors. Perturbation factors are normalized to the corresponding values obtained at the reference field size. Note the different scales of the *y*‐axes.

Figure 5 presents the Monte Carlo calculated relative volume and density perturbation factors for the sensitive volume of the investigated silicon diode detectors SunSILICON and SunSILICON P as a function of the radiation field size of a 6 MV and a 10 MV photon beam. The perturbation factors $p_{\mathrm{vol}}$ and $p_{\rho }$ are shown only for field sizes up to 7 cm, as their variation from unity is within statistical uncertainty for larger field sizes. The values are normalized to the perturbation factor at the reference field size 10 cm. The left panel of Figure 6 shows the remaining perturbation factors $p_{\mathrm{fl}}\cdot s_{w,\mathrm{Si}}$, $p_{\mathrm{chip}}$, and $p_{h}$ of the unshielded silicon diode SunSILICON—all values relative to the corresponding values at the reference field size. The high values of $p_{\mathrm{fl}}\cdot s_{w,\mathrm{Si}}$ are compensated by the silicon chip and housing perturbation factors, which leads to a relatively small field output correction factor $k_{Q_{\mathrm{clin}},Q_{\mathrm{ref}}}^{f_{\mathrm{clin}},f_{\mathrm{ref}}}$ at very small field sizes for the SunSILICON (see Figure 3). While the perturbation factor $p_{chip}$ only deviates significantly from one in very small radiation fields, $p_{fl}$ changes almost linearly with field size. This can also be seen in the right panel of Figure 6 over a larger range of field sizes. Moreover, right panel of Figure 6 shows a comparison between the perturbation factor for the detector housing of the unshielded SunSILICON and the shielded SunSILICON P.

## 4 DISCUSSION

### 4.1 Field output correction factors

Experimental $k_{Q_{\mathrm{clin}},Q_{\mathrm{ref}}}^{f_{\mathrm{clin}},f_{\mathrm{ref}}}$ data is subject to an increased measurement uncertainty at the smallest field sizes, which is mostly due to geometric uncertainties with respect to positioning of the detector and the collimation system of the linac. A thorough discussion on uncertainties associated with small field dosimetry is held in IAEA's TRS 483.^1^ The precautions taken to minimize measurement uncertainty at field sizes less than 3 cm included re‐centering of the detectors for each collimation setting, measurement of the dosimetric field size for each setting of the collimation system, cross‐validation of reference measurement corrections, and cross‐validation of measurements conducted by different co‐authors using different water phantom and detector combinations. Despite that, a residual variation of measured output factors is associated with intra‐type variations of the linacs, which was investigated by Ghazal *et al*..^57^ The group observed a machine related intra‐type variation of output factors of up to 6% at 0.5 cm field size and 1% for field sizes larger than 1 cm for Varian TrueBeam linear accelerators. Their findings agree with the spread of measured values seen in Figures 3 and 4, where these values originate from Varian TrueBeam and Elekta Versa HD linacs.

The experimental data collected with four different SunSILICON and four different SunSILICON P detectors at four different sites is consistent with Monte Carlo simulations with an average root mean square error of 0.003 across the rated range of field sizes of the respective detector types. No significant intra‐type‐variation could be observed between SunSILICON and SunSILICON P models.

At field sizes of less than 10 cm, $k_{Q_{\mathrm{clin}},Q_{\mathrm{ref}}}^{f_{\mathrm{clin}},f_{\mathrm{ref}}}$ values for flattened and unflattened beams are in good agreement for both investigated silicon diode detectors (Figures 3 and 4), which is in line with the data presented in TRS 483. As evident in Figure 4, the shielding component of SunSILICON P extends this property to larger field sizes. Consequently, the data presented in Table 2 is valid for either beam configuration. Notably, $k_{Q_{\mathrm{clin}},Q_{\mathrm{ref}}}^{f_{\mathrm{clin}},f_{\mathrm{ref}}}$ values of the unshielded silicon diode detector diverge for flattened and unflattened beam configurations at field sizes larger than 10 cm (subplot in Figure 3), which exceeds their typical applicable range of use.

TRS 483^1^ recommends against using a detector for field output factor measurements in field sizes, where the necessary correction exceeds 5%, that is 0.95 ≤ $k_{Q_{\mathrm{clin}},Q_{\mathrm{ref}}}^{f_{\mathrm{clin}},f_{\mathrm{ref}}}$ ≤ 1.05. 1048 SunSILICON's $k_{Q_{\mathrm{clin}},Q_{\mathrm{ref}}}^{f_{\mathrm{clin}},f_{\mathrm{ref}}}$ values remain within that range for field sizes from 0.4 to 10 cm (6 MV, 6 FFF) and from 0.5 to 10 cm (10 MV, 10 FFF), respectively, see Table 2 and Figure 3. For most field sizes, the correction value is much smaller. $k_{Q_{\mathrm{clin}},Q_{\mathrm{ref}}}^{f_{\mathrm{clin}},f_{\mathrm{ref}}}$ is within 0.98 and 1.02 for field sizes from 0.6 to 10 cm (6 MV, 6 FFF) and from 0.8 to 10 cm (10 MV, 10 FFF), respectively.

1049 SunSILICON P's $k_{Q_{\mathrm{clin}},Q_{\mathrm{ref}}}^{f_{\mathrm{clin}},f_{\mathrm{ref}}}$ values remain within 0.95 and 1.05 for field sizes from 1.2 to 40 cm (6 MV, 6 FFF) and from 1.5 to 40 cm (10 MV, 10 FFF), respectively, see Table 2 and Figure 4. $k_{Q_{\mathrm{clin}},Q_{\mathrm{ref}}}^{f_{\mathrm{clin}},f_{\mathrm{ref}}}$ values are within 0.980 and 1.005 for field sizes from 2.5 to 40 cm (6 MV, 6 FFF) and from 3.0 to 40 cm (10 MV, 10 FFF), respectively.

The reference data sets collected with the Exradin W1 scintillator and the corrected microDiamond measurements matched and were validated using the data sets collected with the CC04 ionization chamber and the EDGE Detector. The latter two were not used for determining the fit functions, due to the magnitude of the applicable corrections in the field sizes of interest.

### 4.2 Perturbation factors

Previous studies have shown that for field sizes smaller than approximately 3 cm, lateral secondary electron equilibrium is not established at the center of the photon field and that, consequently, the properties of the detector materials affect dose absorption^11^, ^31^, ^58^: When the detector material density is higher than that of water, the dose absorption increases.^8^, ^10^, ^12^ The relative density perturbation factor $p_{\rho }$ (Equation 9) shown in Figure 5 confirms this behavior, which, by itself, leads to an overestimation of dose. Notably, the group of Fenwick et al.^8^, ^10^, ^11^, ^12^, ^14^ and Benmakhlouf & Andreo^7^, ^9^ investigated the roles of mass and electron densities, *i*‐value, and density effect and discussed different approaches to interpreting and analyzing their impact in small field measurements.

The key difference in their approaches lies in the different segmentation of $s_{w,\mathrm{Si}}\cdot p_{\mathrm{fl}}\cdot p_{\rho }$ (see Equations 8 and 9). Both groups propose the use of a fictitious intermediate material with a density of 2.33 g/cm^3^. While Benmakhlouf & Andreo then apply $s_{w,\mathrm{Si}}$ corresponding to that of 2.33 g/cm^3^ water,^7^, ^9^ Fenwick *et al*. apply $s_{w,\mathrm{Si}}$ corresponding to that of 1 g/cm^3^ water,^8^, ^11^ which is the approach adopted here (see Figure 2). While $s_{w,\mathrm{Si}}$ and density are linked in real materials and the interpretation of the resulting factors may differ, Fenwick et al. showed that the p‐values determined with both methods are within 0.3%.^8^

The perturbation factor $p_{\rho }$ observed in the smallest investigated radiation field (0.6 cm) deviates by less than 0.5% from unity. The underlying reason was discussed by Fenwick et al.,^11^ who demonstrated that density perturbation decreases with the thickness of the sensitive volume in the direction of the beam. The thickness of the sensitive volume of the silicon diodes used in this study is only 30 µm.

Volume perturbation of the absorbed dose also becomes increasingly prominent in very small fields. In contrast to the density perturbation factor $p_{\rho }$, however, volume perturbation $p_{\mathrm{vol}}$ (Equation 10) leads to an underestimation of dose because of averaging within the detector's sensitive volume. Figure 5 also illustrates the relative volume perturbation factor $p_{\mathrm{vol}}$ for the diode used in SunSILICON and SunSILICON P. Evidently, the volume perturbation factor $p_{\mathrm{vol}}$ slightly overcompensates the diode's density perturbation factor $p_{\rho }$. The combined effect reduces the impact of perturbations introduced by the inactive region of the silicon chip $p_{\mathrm{chip}}$ (Equation 7) and the detector housing $p_{h}$ (Equation 6) surrounding the sensitive volume, which are shown in the left panel of Figure 6. Notably, the impact of the unshielded SunSILICON detector's housing component is negligible except for the smallest field sizes. This can be explained by the SunSILICON housing consisting mostly of water‐equivalent HE Solid Water (Sun Nuclear Corp.) and near‐water equivalent epoxy.

While $p_{\rho }$ and $p_{\mathrm{vol}}$ characterize the impact of the sensitive material's density and volume, the product $p_{\mathrm{fl}}\cdot s_{w,\mathrm{Si}}$ describes the influence of its atomic composition (Equation 8) and *I*‐value. $p_{\mathrm{fl}}\cdot s_{w,\mathrm{Si}}$ characterizes the dominant effect in the dose deposition in the sensitive volume, which has a strong field size and energy dependence, as seen in Figure 6 (both panels). At lower beam energies, the relative product of $s_{w,\mathrm{Si}}$ and $p_{\mathrm{fl}}$ deviates further from unity (Figure 6, right panel).

Since the stopping power ratios $s_{w,\mathrm{Si}}$ of water and silicon exhibit only minor variations with field size,^59^ the fluence perturbation factor $p_{\mathrm{fl}}$ can be identified as the source of the field size dependence of $p_{\mathrm{fl}}\cdot s_{w,\mathrm{Si}}$. The fluence perturbation factor $p_{\mathrm{fl}}$ thus emerges as the dominant contributor to the observed over‐response in large photon fields (Figures 3 and 6, right panel) and under‐response in smaller photon fields (Figures 3 and 6, left panel) relative to the reference field size of 10 cm. Since $p_{\mathrm{fl}}\cdot s_{w,\mathrm{Si}}$ is a material property of silicon, this property is shared among all unshielded silicon diode detectors,^1^ including SunSILICON. Hence, unshielded silicon diode detectors typically have a maximum field size specification.

The impact of $p_{\mathrm{fl}}$ observed at smaller field sizes is partially compensated by the perturbation effects introduced by the detector housing $p_{h}$ and the inactive volume of the diode chip $p_{\mathrm{chip}}$, which become dominant at the smallest field sizes. Their influence, however, diminishes at field sizes larger than 4 cm × 4 cm, where $p_{h}$ defines the detector behavior (compare Figure 3, subfigure, and Figure 6, right panel).

The importance of the shielding component design on the detector response in large photon fields is illustrated in the right panel of Figure 6, where the perturbation factors of the shielded housing, $p_{h,\mathrm{sh}.}$, and the product $p_{\mathrm{fl}}\cdot s_{w,\mathrm{Si}}$ display nearly opposite trends for both beam energies and field sizes of about 4–40 cm. The observed energy dependence of $k_{Q_{\mathrm{clin}},Q_{\mathrm{ref}}}^{f_{\mathrm{clin}},f_{\mathrm{ref}}}$ in that field size range is within measurement uncertainty, or statistical uncertainty, respectively (Figure 4). On the other hand, the shielding component leads to a significantly enhanced detector response in field sizes smaller than 2 cm. This trade‐off is common in shielded silicon diode detector designs, which often have a minimum field size specification.

## 5 CONCLUSION

This study presents the first comprehensive dataset—using both Monte Carlo simulations and experimental measurements—on photon field output correction factors for the novel unshielded and shielded silicon diode detectors SunSILICON and SunSILICON P. The results demonstrate that both detector types are suitable for clinical dosimetry across a wide range of field sizes and provide an in‐depth analysis of the underlying physical phenomena.

It was shown that the dose response behavior of silicon diodes across varying field sizes is predominantly governed by the fluence perturbation factor $p_{\mathrm{fl}}$, which reflects the influence of the sensitive material's atomic composition (silicon). The interplay of fluence perturbation $p_{\mathrm{fl}}$, the detector housing perturbation $p_{h}$, and the perturbation of the inactive chip volume $p_{\mathrm{chip}}$ minimizes the combined magnitude of perturbation effects, making the unshielded silicon diode detector SunSILICON suitable for small field dosimetry with only minor corrections.

Our work demonstrated that the shielding component of the silicon diode SunSILICON P compensates for the silicon‐induced fluence perturbation $p_{\mathrm{fl}}$, which dominates at large photon field sizes. The resulting field output correction factors $k_{Q_{\mathrm{clin}},Q_{\mathrm{ref}}}^{f_{\mathrm{clin}},f_{\mathrm{ref}}}$ are near unity in large photon fields.

The present work enhances understanding of these detectors with regard to their suitability for output factor measurements.

## AUTHOR CONTRIBUTIONS

Andreas A. Schönfeld led the detector design, drafted the manuscript, supervised the study, and conducted data acquisition (Monte Carlo and experimental) as well as data analysis. Mohamad Alissa contributed to the Monte Carlo simulations. David Towle, Andy Murray, Ann‐Britt Schönfeld, Erik Alquist and Charbel Habib conducted the measurements on the Varian linac. Teresa J. Anders and Gerhard Wessing conducted the measurements on the Elekta linac. Olivier Evrard and Jeff Hildreth designed and tested the diode. Chuck Rosenwald contributed to detector design and experimental studies. Veronika Flatten contributed to the design of the study and analysis. Damian Czarnecki lead the Monte Carlo simulation study and data analysis, as well as substantially revised the manuscript. All authors revised the manuscript and approved the final version.

## FUNDING INFORMATION

This research received no external funding.

## CONFLICT OF INTEREST STATEMENT

Andreas A. Schönfeld, David Towle, Andy Murray, Ann‐Britt Schönfeld, Erik Alquist, Olivier Evrard, Chuck Rosenwald, Jeff Hildreth, Veronika Flatten are employees of Mirion. The remaining authors declare that the research was conducted in the absence of any commercial or financial relationships that could be construed as a potential conflict of interest.

## ETHICS STATEMENT

This study did not involve human participants or animals. All experiments were performed using phantoms and simulation data; therefore institutional review board approval and informed consent were not required.

## Figures and Tables

**FIGURE A.1:**
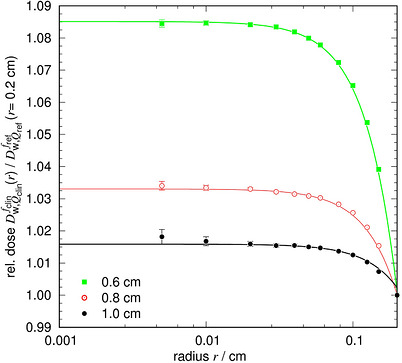
Monte Carlo calculated dose in a cylindrical water voxel with a height of 0.2 cm as a function of the radius r normalized to the dose in a cylinder with height of 0.2 cm and radius of 0.2 cm. The statistical uncertainty of the Monte Carlo–calculated values is indicated by error bars, which are predominantly smaller than the symbol size. The magnitude of statistical uncertainties decreases with increasing radius of the scoring volume.

## Data Availability

The data that support the findings of this study are available from the corresponding author upon reasonable request.

## 1

The absorbed dose at a point $D_{w,\;Q_{clin}}^{f_{clin}}$ can only be determined indirectly when using Monte Carlo simulations by scoring the mean dose within a voxel of finite size. This limitation is distinctly critical in small‑field dosimetry, where volume‑averaging effects and the choice of scoring voxel size can significantly influence the accuracy of the calculated dose $D_{w,\;Q_{clin}}^{f_{clin}}$. This effect is particularly pronounced with respect to the lateral extent of the scoring volume perpendicular to the beam axis. For this reason, we determined the dose in a cylindrical voxel with a fixed height of 0.2 cm while varying the cylinder radius r. The cylindrical shape was chosen to take advantage of the anisotropic dose gradients in small fields: while lateral dose gradients vary with field size, the depth dose gradient is nearly linear at reference depth. By approximating the point dose with a cylinder rather than a sphere, the calculation efficiency can be improved.

The absorbed dose to water as a function of the scoring‑volumes radius 𝑟, normalized to the dose in a cylinder of 0.2 cm radius, is shown in Figure A.1 for field sizes of 0.6 cm, 0.8 cm, and 1 cm. For small radii, the dose $D_{w,\;Q_{clin}}^{f_{clin}}$ as a function of r can be approximately fitted to a second‑degree polynomial, as shown in Equation 2. By extrapolating to r=0 cm the absorbed dose at the reference point can be estimated.
